# A systemic approach to identifying sustainable community-based interventions for improving adolescent mental health: a participatory group model building and design protocol

**DOI:** 10.1186/s12961-024-01247-y

**Published:** 2025-01-13

**Authors:** Megan Keenan, Leanne Freeman, Ediane Santana de Lima, Katie Potter, Tim Hobbs, Ellis Ballard, Peter Fonagy

**Affiliations:** 1 Dartington Service Design Lab, University College London, London, United Kingdom; 2 Dartington Service Design Lab, Buckfastleigh, United Kingdom; 3 Brown School at Washington University in St. Louis, St. Louis, United States of America; 4 University College London, London, United Kingdom

**Keywords:** Mental health, Children and young people, Community-based system dynamics, Human-centred design, Social determinants, Place-based

## Abstract

**Background:**

The deteriorating mental health of children and young people in the United Kingdom poses a challenge that services and policy makers have found difficult to tackle. Kailo responds to this issue with a community-based participatory and systemically informed strategy, perceiving mental health and well-being as a dynamic state shaped by the interplay of broader health determinants. The initiative works to explore, define and implement locally relevant solutions to challenges shaping the mental health and well-being of young people. Kailo unfolds in three stages within each locale. These stages encompass: “early discovery”, “deeper discovery and co-design” and “implementation”. This document delves into the participatory group model building and design protocol occurring in the “deeper discovery and co-design” stage of the project.

**Methods:**

Participatory methods, such as group model building, are effective in articulating and building consensus on complex issues like the social determinants of adolescent mental health. This paper describes the protocol for application of group model building within the Kailo design process to develop causal loop diagrams and pinpoint leverage points for improving adolescent mental health. It also suggests a method for considering modifications to delivery within a unique project context and in alignment with participants’ needs. This paper sets out to define the approach and clarify the objectives these engagements aim to fulfil. The method adapts existing group model building (GMB) protocols for use in a community setting. The engagements will involve groups of local young people and existing community members. To assess the success of the session’s implementation post-delivery, the study utilizes existing frameworks for fidelity evaluations, which define a core and flex model.

**Discussion:**

The method described enables an integration of diverse local understandings of complex processes which provides a platform for creating co-designed interventions. This protocol can be used to further strengthen research and design through incorporating complexity and participation into the formulation of contextually relevant policies and practices. The strengths and limitations of the approach are discussed.

## Introduction

This paper addresses adolescent mental health and wellbeing as a dynamic and systemic concern and introduces a research and design framework named Kailo that facilitates local partnerships in devising systemic, youth-centred and evidence-based solutions to local challenges. The principal focus of this protocol paper is to outline the rationale and methods for a series of engagements using a participatory group model building approach within in a co-design process to identify the systemic drivers of mental health and well-being for young people and support the development of locally relevant solutions. It clarifies the reasons for selecting these methods, provides details on the methods employed in the sessions, adaptations for the application of group model building with young people and establishes criteria for evaluating the success of the sessions in relation to their objectives. In doing so, it also seeks to record an approach to participatory group model building in youth-led community settings, expanding the reach of complexity thinking to populations with lived experience of social challenges. A subsequent paper will then detail adherence to the protocol, where modifications might be necessary, and the results and outcomes of the process.

## Background (problem context)

In the United Kingdom, an expanding body of evidence highlights a persistent decline in children and young people’s well-being and mental health across recent decades [1, 2]. This trend persists despite investments in public health and social care [3], revealing that services struggle to meet the growing demand [4].

Historically, these investments have favoured mental health models that mirror pathological models of physical health, advocating for individualized treatment [5]. As a result, services, interventions and therapies predominantly target diagnosed mental disorders, neglecting a holistic model of mental well-being that includes a variety of social, individual and community factors [6]. Furthermore, public health initiatives aimed at tackling the sociological determinants of mental health often focus on macro-level factors, overlooking the intricacies of local contexts [7]. The dynamic and multifaceted nature of this issue, coupled with the diversity of system stakeholders and their differing objectives, presents a complex systemic challenge [8]. This complexity underlines the need for a community-focused, complexity-aware approach to understanding the local factors contributing to the decline in adolescent mental health.

## The Kailo programme (programme context)

### Background

**Fig. 1:**
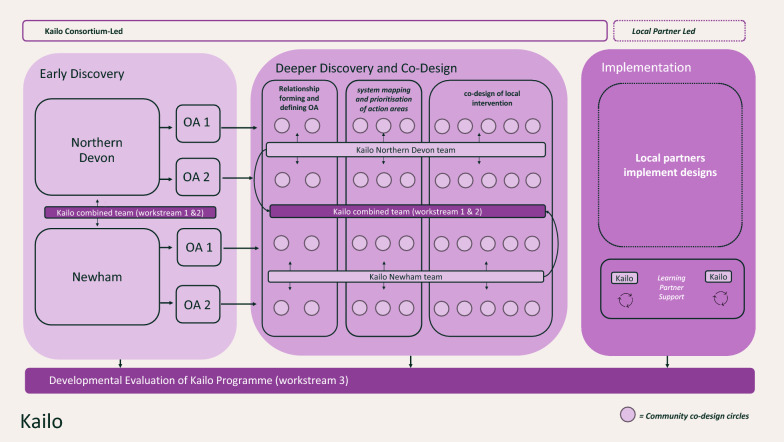
The Kailo implementation framework

In response to the necessity of adopting a systemic perspective to tackle the social determinants of young people’s mental health, our research group has developed and is executing “Kailo”. This is a 5-year research and design programme financed by the UK Prevention Research Partnership (UKPRP). Kailo is a structured framework that assists local partnerships in exploring the specific social and environmental factors affecting young people’s mental health, identifying priorities and then collaboratively designing youth-centred, evidence-based policy and practice solutions [9]. It is initially being applied in the London borough of Newham and the rural region of Northern Devon in the Southwest United Kingdom: two distinctly different areas intentionally chosen to examine the impact of local context on young people’s mental health and wellbeing. The Kailo framework encompasses three main phases in the local regions—“early discovery”, “deeper discovery and co-design” and “prototyping, implementation and testing” (see Fig. 1) [9]. The implementation of these phases are carried out by local Kailo teams in each location (workstream 1) and are supported by research experts throughout the programme (workstream 2). Alongside the development and execution of the framework, an evaluation team (workstream 3) is performing a theory-based formative evaluation to examine the presuppositions of the underlying programme theory of Kailo [10].

### Early discovery phase of Kailo

The primary goal of the early discovery phase is to comprehend the local factors and priorities affecting young people’s mental health from a social determinants perspective [11]. During this stage, researchers collaborate with local stakeholders to identify and prioritize specific areas of concern that both the community and the Kailo team believed could be effectively tackled (ibid). This involves qualitative engagements with a wide range (500+) of young people and community professionals across Northern Devon and Newham [11].

This stage also includes a scoping review of existing literature on the social determinants of adolescent mental health. This includes systemic and interdependent factors contributing to young people’s mental health and well-being in the UK, such as poverty, housing, transport, school exclusion, social connection, discrimination, safety, knowledge and norms related to mental health, future opportunities and sense of belonging [12, 13]. This body of knowledge works to further to establish a shared understanding of local priorities, including areas where needs might be intensified.

These qualitative engagements, combined with a literature review, nurture a mutual understanding of local priorities and avenues for “systemic change” [14]. The outcomes are delineated as opportunity areas (OAs) for systemic change concerning social determinants of young people’s mental health and well-being (such as “violence and crime”, “mental health literacy” and “jobs and opportunities”). These identified opportunity areas are then agreed upon to be advanced to the “deeper discovery and co-design” phase of Kailo.

### Deeper discovery and co-design

The second phase of the Kailo framework—the deeper discovery and co-design phase—utilizes a community-based participatory approach involving a diverse group of stakeholders with varied lived, professional and academic experiences. By amalgamating this wide array of knowledge and experience within local systems, the programme enables communities to devise meaningful, sustainable enhancements in young people’s mental health and wellbeing [15].

The deeper discovery and co-design phase capitalizes on a variety of methodologies, including rapid realist reviews of evidence, participatory action research led by young peer researchers and community partners, community-based system dynamics and creative design methods from the fields of user-centred design and “design thinking” [16–19].

This phase employs these techniques to develop and trial strategies that address the systemic drivers of young peoples’ wellbeing and mental health and engage and collaborate with key community stakeholders to bridge gaps in engagement, knowledge and created shared visions. In doing so, it supports the design of locally relevant and sustainable solutions. Furthermore, this phase works to enhance local capacity for transferring ownership of interventions from the Kailo team to community-led partnerships [11].

Further information on deeper discovery will be detailed in an upcoming paper.

### Implementation: testing, embedding and learning

The final stage of the Kailo framework—prototyping, implementation and testing—follows from the deeper discovery and co-design process. The objective of this phase of work is to facilitate local system integration, prototyping and iterative refinement of the interventions developed in the previous phase of work [9]. This will be achieved through three rounds of “low fidelity” prototyping and testing of interventions, followed by subsequent rounds of “high fidelity” sustained implementation of local designs [9]. Within this testing process, the Kailo team will also endeavour to transfer ownership of the design process to local system leaders. Ultimately, this phase of work is designed to support interventions to become locally embedded and sustained, enabling them to enhance youth mental health and wellbeing outcomes within the local area [9].

## Methods

### Group model building within in the Kailo framework

Kailo stands out by adopting a systemic, evidence and community-informed approach to identify, understand and design interventions to enhance the social determinants of young people’s wellbeing and mental health. Whereas traditional research methods often concentrate on a limited set of factors and perceive relationships between variables as linear, systemic frameworks facilitate the recognition of interconnected, dynamic relationships among a broad array of factors [20, 21]. This fosters a more comprehensive understanding of systemic challenges, enabling various system actors to implement actions that tackle the systemic drivers of these challenges [21].

The distinctiveness of this approach is supported by the incorporation of participatory group model building (GMB) within the deeper discovery and co-design phase of the Kailo framework. Group model building (GMB) is a methodology grounded in the discipline of system dynamics that combines the tenets of systems thinking with participatory techniques to tackle complex issues [22]. GMB aids in the identification and illustration of both underlying causes and sequences of causation leading to social problems. This enables participants, researchers and broader system stakeholders to gain a clearer understanding of how initiatives can spark change and assists in the prioritization of areas for action for the co-creation of community-based interventions within the Kailo framework [22].

While participatory applications of group model building, such as community-based system dynamics, are well-established in similar contexts, their application with young people, though increasing, remains rare [23]. GMB has been deployed in research contexts akin to the Kailo project, encompassing studies on social determinants of health [24], adolescents’ perspectives on public health in the United Kingdom [22], the formulation and influence of local policy [25] and forthcoming research on youth mental health [26].

### Rationale for group model building

#### Why group model building?

GMB was selected as a key method for the Kailo deeper discovery and co-design phase because it provides a structured and collaborative means to engage with a variety of participants. It aids in elucidating the underlying system dynamics and drivers of the issues at hand and can pinpoint potential interventions or leverage points for changes in policy or practice. Stemming from system dynamics, GMB seeks to depict a system by drawing on the diverse perspectives of stakeholders. The core principle of GMB is that capturing a comprehensive understanding of a systemic problem necessitates the amalgamation of various mental models of the issue [27]. While it has traditionally utilized quantitative methods, GMB now increasingly incorporates qualitative, participatory techniques for modelling systemic problems [28], as demonstrated by community-based system dynamics (CBSD) [16]. When applied to young people, GMB can unveil context-specific systemic drivers of a problem they find most pertinent. Through this approach, it develops and clarifies a collective hypothesis of the connections between these drivers [21]. For these reasons, GMB works to provide greater depth to the ‘definition’ and ‘developing’ phases of a traditional design process and supports identifying interventions that are tailored to respond to the complex realities of local contexts [29, 30].

#### Core components of group model building

GMB sessions usually adhere to pre-defined scripts that are accessible on open-source platforms like Scriptapedia [31]. These scripts are organized by the purpose of the activity (presentation, divergent information, convergent information and evaluation) and are distinguished as either “established” or “promising”.

Workshop outcomes are often synthesized and refined in an iterative manner (see [32]). This refinement may occur if participants were uncertain about the modelling activity, encountered difficulties in diagramming during the session, mentioned an element not captured in the model, or changed the meaning of a variable during modelling. Any adjustments to the model by the research team are then shared with the group for feedback or review [16].

Group model building functions through two main mechanisms: the models it produces and the collective modelling process itself [33]. This approach promotes team learning, fosters consensus on systemic challenges, ensures multi-stakeholder commitment to action and reveals power dynamics from which diverse perspectives emerge [34]. As a qualitative modelling method, participatory group model building is well-suited for exploratory research into systemic issues. It has proven effective in developing a shared understanding of a systemic problem, providing deeper insights into and agreement on points of intervention and impacting both local and national policy shifts and interventions [33, 35].

In the subsequent sections of this paper, we will outline the participatory group model building approach that will be applied for Kailo. In doing so, we will discuss the rationale for participatory group model building; provide an overview of the aims, participants, settings, facilitation team, ethics and considerations pertinent to the GMB context, detail the planned approach and session activities, considerations for working with young people and consider the evaluation and measures of success related to the GMB process. Finally, the discussion will elaborate on the distinctiveness of the approach, modifications related to core and flexible elements of participatory GMB and any potential risks from the research design and process.

### Group model building for Kailo: overview

#### Aim

In alignment with the objectives of the deeper discovery, the group model building sessions aim to:

- Engage and collaborate with key stakeholders and actors related to the identified opportunity areas, encompassing young people, youth and community organizations, local commissioners and other pivotal actors;
- Develop a more detailed understanding of the prioritized opportunity areas as delineated and experienced by young people and the wider local communities; and
- Acquire a more thorough understanding of the systemic behaviours influencing the identified opportunity areas and identifying leverage or intervention points for meaningful change.

#### Participants

Workshop participants for the deeper discovery phase consist of a carefully chosen group of 16–20 local system stakeholders, called the “small circle”, with a particular emphasis on reflecting the lived experience of young people. This emphasis on lived experience aids both the conceptualization of the systemic challenge from the viewpoint of those encountering the challenge and enhances the validity of the session outputs [36]. Local organizations, practitioners and community members who have pre-existing relationships with young people with lived experience pertinent to the opportunity area and mental health are engaged to support recruitment for the “small circle”. The session recruitment also prioritizes young people with representation from lesser heard or marginalized voices [28]. Representatives from local partners are also engaged throughout the sessions to encourage and facilitate young people’s participation, which is vital for ensuring diverse representation and fostering meaningful involvement in the co-design process.

The “small circle” is designed to remain the same throughout the sessions to preserve a continuity of perspectives and guarantee a developing understanding of the matters at hand.

Participants are compensated for their time in the sessions and have travel expenses covered in acknowledgment of their expertise and contribution to deeper discovery.

#### Setting

The deeper discovery and co-design workshops take place in community venues provided or secured through local community partners (such as youth clubs or community centres) in both Newham and Northern Devon. These venues are chosen because of their familiarity to many of the young people participating in the sessions; their use signifies the ongoing commitment of local partners and, practically, ensures they can be consistently available for sessions. Community partners chosen to host the workshop sessions are also included in the development and facilitation of the workshops to offer additional familiarity and continuity between workshop sessions.

#### Facilitation team

The facilitation team primarily consists of individuals involved in leading the entire “deeper discovery and co-design” phase of Kailo (workstream 1). This allows the team to leverage existing relationships established within the local area and among the workshop participants and aids in advancing the co-design process by integrating insights from the GMB workshop sessions. Regular meetings with the lead facilitators, researchers (including young researchers, see Table 1) and system experts across the local areas are organized to exchange learnings across sites throughout the implementation phase. Additionally, a member of the Kailo evaluation team participates in each workshop session to contribute to the formative evaluation of the Kailo framework [9]. This evaluation team member is not part of the facilitation team.

**Table 1:** facilitation team roles and responsibilities

Role	Tasks
Meeting convenor and closer (site co-lead)	Primarily responsible for initiating the session, welcoming participants to the exercise, ensuring that participants comprehend the purpose of the exercise within the context of their organization or community and introducing the facilitators. This individual also concludes the session and express gratitude to participants for their time This is someone who already maintains an established relationship with the group (such as the Kailo site lead for each local area)
Community facilitator(s)	The community facilitator’s main responsibility is to utilize their social capital to assist the community in collaborating with the modeller. facilitator(s). This individual is well-acquainted with the local understanding of the problem being modelled and is familiar with community norms. The facilitator is equipped with basics on group facilitation and some exposure to system dynamics through the planning process and training session or workshop. For the Kailo project, this includes young researcher(s) and community partners. Young researchers (16–25 years of age) are recruited from the local area into the Kailo site team. They may also have lived experience with the opportunity areas discussed. Where feasible, the facilitation team includes community partner(s) who have pre-established relationships with the young participants—such as youth workers or mentors.
Modeller facilitator	Bears the main responsibility for system dynamics modelling and group model building process. This individual is skilled in systems thinking/system dynamics modelling with expertise in instructing and guiding groups in the application of community-based system dynamics. The person also has experience in facilitating groups and leading group model building sessions. The modeller facilitator(s) is also tasked with introducing the rest of the facilitation team to the work—including basic training on system methods employed for each session. They may also utilize outputs from the group session to design or digitize learning (such as by converting handwritten causal loop diagrams into digital formats using Stella Architect modelling software). This can involve refining causal mechanisms to reflect the behaviour described during the sessions, to be shared back with the group.
Production coordinator/note taker	The production coordinator’s main responsibility is to ensure that the information gathered during the exercises, including diagrams, notes, electronic versions of diagrams, etc., are collected, appropriately archived and made accessible. This involves noting major themes, points of discussion, etc., that might be overlooked by the modellers

Prior to the GMB workshops, the facilitation team receives training in core concepts of systems thinking and group model building. This equips the team to fulfil their roles within the workshop sessions. The core modeller facilitators also develop facilitation guides adapted from Scriptapedia to support the choreography of each session [31].

During the workshops, the facilitation team assumes different roles to ensure the smooth execution of the process (see Table 1). These roles, adapted from the open-source online resource, Scriptapedia [31], aim to ensure the workshops are productive, inclusive and focussed, allowing for effective exploration of the systemic drivers of local opportunity areas/priorities.

#### Ethics and safeguarding

The psychological safety and trust of participants taking part in the workshop sessions is paramount to the research process. This is supported through a comprehensive safeguarding procedure with designated safeguarding leads, confidentiality and data agreements and a working group agreement developed within the small circle sessions. One-to-one interviews with young people and the lead facilitators are also conducted to allow for any additional needs to be raised before engaging in the workshops. Additionally, a confidential feedback mechanism is provided to ensure that young people can submit feedback about any concerns or sensitive topics that may arise within the sessions.

The facilitation and research team is also trained in safeguarding and trauma-informed practice as a part of the overarching Kalio programme to ensure the safety of participants and researchers throughout the Kailo process.

#### Considerations

Given the variety of opportunity areas Kailo facilitation team experience, and differences in local contexts, some variation in participants and the facilitation team roles is anticipated. A degree of flexibility and adaptability is essential for operating within a highly participatory and community-focused setting. Additionally, working with young people necessitates extra considerations and potential adaptations to ensure proper safeguarding measures are implemented. Where such adaptations may occur, the Kailo team will aim to prioritize firstly the safeguarding of young people and secondly achieving the core objectives of deeper discovery and co-design outlined above.

### Engagement design

**Fig. 2:**
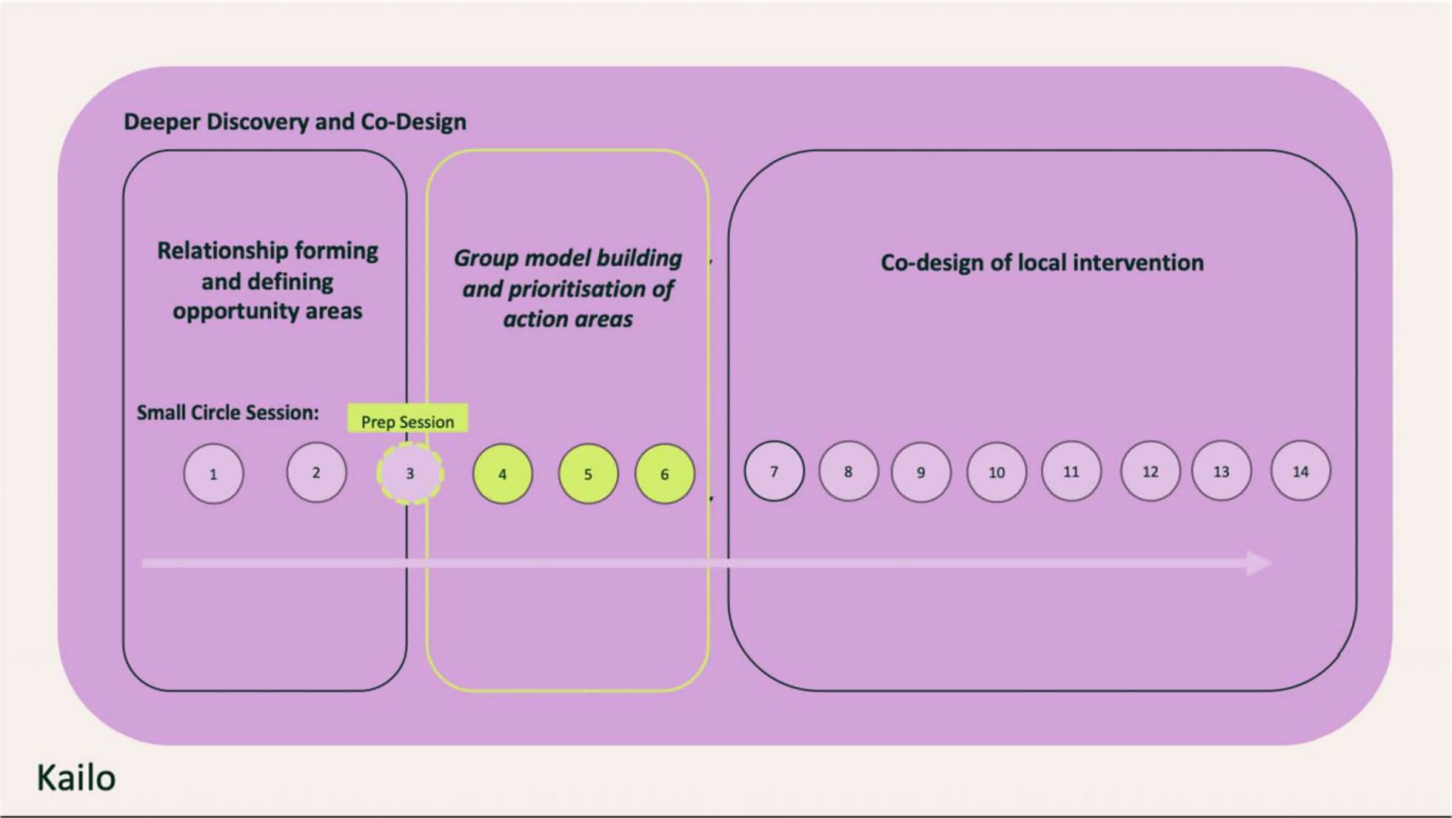
Group model building within deeper discovery and co-design

The group model building sessions unfold over four workshops with the co-design groups in community venues. The workshops are scheduled for 2–3 h on weekday evenings to accommodate the schedules of young people engaged in college or work (Fig. 2).

Each engagement includes an introduction to the session, followed by a series of activities introduced through plenary input and then undertaken in small, facilitated groups.

#### Sessions and activities

There are four sessions, comprising one preparation session and three GMB sessions, which involve systems activities and the attendance of the modeller (see Table 3). To optimize time in the sessions, one group model building activity—hopes and fears—is conducted in the preparatory small circle session before the main sessions start. This provides participants with the chance to express their initial visions for systemic change and any concerns that might need addressing in the sessions [37].

The workshops are structured to build upon one another, following a structured collaborative process that leads participants through the steps necessary to develop causal loop diagrams (CLDs) [16] and pinpoint leverage points for systemic change [21]. Between workshops, the facilitation team led by the modeller synthesizes and examines some of the insights from the previous workshop. This work is then shared at the start of the subsequent workshop, and the outputs are distributed around the room to be expanded upon with the new activities.

See “Appendix” for detailed facilitation guides.

#### Participatory group model building tools

Participatory group model building tools are essential for facilitating the process of systems change work. Each tool serves a distinct purpose within the broader process.

As detailed in Tables 2 and 3, each workshop generates a variety of outputs from the activities. Any discussion from participants not captured through the activities is supplemented with facilitator notes. These outputs are then reviewed and synthesized by the facilitation team, led by the modeller, between sessions using thematic analysis and system dynamics principles as follows:

- Workshop 1: Identifying key, connected variables and any emergent feedback loops.
- Workshop 2: Constructing causal loop diagrams, including identifying additional feedback loops, multi-loop structures, and potential leverage points.
- Workshop 3: Reviewing prioritized feedback loops in the integrated system map.

**Table 2:** An overview of the systems sessions’ aims and activities

Session	Aims	Activities
Prep session	Fosters relationship development, establishes a grounding in the vision for change and surfaces any concerns that may need addressing in GMB sessions	Hopes and fears [37]
(1) Co-defining the opportunity area	Develops an understanding of the historical behaviour and systemic contributors to the specific opportunity areas	Graphs over time [43] Variable elicitation [37] Connection circles [44]
(2) Exploring connections	Identifies and explores the connections within the system to uncover the underlying behavioural mechanisms driving the “opportunity area” for change Surfaces and negotiates various mental models from the different stakeholder groups (including young people, community organizations and academic researchers)	Causal loop diagrams[45]
(3) Identify leverage points and action areas for systemic change	Investigates the connection between existing resources within the system and impact areas to feed into co-designing local interventions with key system stakeholders	Places to intervene [46] Action Ideas [46]

**Table 3:** Overview of participatory group model building scripts

Script name	Description	Output
Hopes and fears	This affinity sorting exercise surfaces group expectations and aspirations regarding future changes. It establishes group expectations about the system change work and envisions what the change can resemble. This exercise is crucial for broadening and shifting underlying mental models about the systemic issue [37]	List of participants hopes and fears
Trends over time	These are simple line graphs that illustrate the pattern of change for a specific variable or systemic issue over time. The graphs are useful for defining boundaries around the opportunity area, narrowing the scope, audience and timeframe of an issue [44] Graphs over time are also beneficial for developing an understanding of a problem through a systemic lens. Complex, or systemic, issues are challenges that relate to every part of a system. These are often issues that are dynamic, include multiple stakeholders with different objectives, involve time delays between action and outcome, provoke unintended consequences and have accumulations or a history of dependence [8]. By considering how a problem has evolved in the past, workshop participants and key system stakeholders are better equipped to think about the factors that may have influenced that change	Graphs of dynamic variable trends related to the opportunity area
Variable elicitation	This tool draws on insights from initial understandings about the systemic challenge at hand [37]. It provides a deeper understanding of the patterns observed in the graph over time activity. By identifying variables related to the systemic challenge and clustering observations into themes, it becomes easier to analyse, map and utilize key insights. The themes identified in the variable elicitation exercise can then be built upon for subsequent system mapping activities as a “word bank”	Prioritized list of themes and variables related to the opportunity areas
Connection circles	Connection circles are a visual tool that can help to identify connections and directionality of relationships between variables endogenous to a system. A connection circle helps stakeholders to uncover some “causal relationships” and begin to consider how these connections create feedback relationships—when the effect of a causal impact comes back to influence the original cause of that effect [44]	Initial identification of causal relationships between variables related to the opportunity area
Causal loop diagram	Causal loop diagrams (CLDs) are a method of conceptualizing and diagramming feedback behaviours in a system to understand and articulate complex system behaviour. Critically, CLDs are a tool for surfacing, visualizing and exploring underlying mental models of stakeholders [28]. Through mapping CLDs, stakeholders are better positioned for identifying points of leverage—places where sustainable change can occur—within the system. They are also an important tool for consensus building and shifting mental models, through negotiating and engaging the perspectives of multiple stakeholders in the system (as defined by the makeup of participants within the workshop sessions)	Causal loop diagram(s) related to the opportunity area
Places to intervene	An advantage of systems thinking and mapping is utilizing an understanding of the structure of a system to consider what interventions, or leverage points, would have the greatest influence on systemic change. Defined by Donella Meadows, leverage points are the “places within a complex system where a small shift in one thing can produce big changes in everything” [46] Placed within the context of local resourcing, the “places to intervene” activity challenges stakeholders to consider the types of interventions that may be utilized to create the changes they are seeking locally. This will build into a prioritization exercise as part of “action ideas” identified below	List of recommended places to intervene within the system related to the opportunity area(s)
Action ideas	Action ideas is an activity aimed at identifying and prioritizing actions after a systems map or model has been created. This is done through mapping intervention ideas onto a cross-sectional grid that ranges from “high impact” to “low impact” and “high resource” to “low resource”. It is a way to translate learning from the insights gathered in the process thus far into action for systems change [46]	Prioritized list of action ideas related to the opportunity area(s)

This is then presented back to the co-design groups for reflections and amendments, and subsequent analysis is carried out through the cumulative activities. Where necessary, the lead modeller works to revise the causal loop diagrams to represent the workshops findings utilizing system dynamics theory (such a system archetypes) [38]. This is always presented back to the co-design group to ensure collective ownership.

Analysis and integration are undertaken by the facilitation team using Miro, an online whiteboard and workshop tool (see [39, 40]). Refined CLD outputs are designed using Stella Architect, a specialist System Dynamics software [16].

A refined causal loop diagram with the identified and prioritized leverage points for each opportunity area is presented to the workshop participants and wider system stakeholders to further discussion and action planning. In this manner, the causal loop diagrams are intended to act as a visualization of the lived experiences and mental models of the workshop participants that can be communicated outwardly to influence action related to addressing young people’s mental health and wellbeing locally.

The prioritized action areas (in accordance with their leverage in the system) are then used as a guide for co-designing interventions within the small circle for the remaining sessions of the deeper discovery phase [11]. This process is intended to support developing interventions that are systemically informed and locally owned. This is a critical component to the Kailo framework for addressing the systemic problem of declining mental health and well-being for young people living in the United Kingdom [1, 2].

#### Considerations for young people

To support the engagement of young people throughout the GMB activities, the sessions are designed with flexibility and opportunities to further inputs outside of traditional GMB scripts. This includes creative inputs (such as drawing a desired hope or fear) or guided discussions with the facilitation team. The sessions also include opportunities for relationship forming, refreshments and energizing activities to promote engagement (see “Appendix” for facilitation guide).

#### Adaptations

Given the evolving outcomes from each workshop, the imperative of safeguarding participants, and the innovative nature of the application, the protocol may undergo responsive adaptations within and between each session and small circle groups [41]. These changes may include alterations to the scripts used in the GMB sessions, adjustments to the duration or sequencing of activities in line with time limitations, and seeking additional feedback from participants outside the GMB sessions. Allowing for adaptability ensures that a broad spectrum of viewpoints is integrated into the model, enriching its depth and relevance.

To ensure that the fundamental mechanisms of group model building are preserved, and that the project’s key outputs are realized, the facilitator team employ an analytical framework. This framework guides them in assessing whether the in-session adaptations maintain the core mechanisms. This assessment is grounded in theoretical insights derived from realist evaluation [42]. Such a structured approach ensures that while the protocol remains flexible and adaptive, it does not deviate from its primary objectives or compromise the integrity of the group model building process. Every alteration made to the protocol is meticulously documented, providing transparency and a clear trail for future reference and replication.

### Evaluation

As part of the overarching Kailo framework, a developmental realist evaluation is conducted to support learning and improvement of the framework. This evaluation enables us to better understand how, why, and for whom, Kailo functions as an initiative; the conditions necessary for place-based systems change to be achieved and the outcomes prioritized in the process [10]. This includes an assessment of the contributions of GMB to achieve the objectives of Kailo. The evaluation also considers the contribution of GMB within the entirety of the three phases of Kailo [9]. This allows for evaluation not only of the success of the individual components of each GMB session and how it works with other methods used within the Kailo framework, but also a consideration of the wider contribution of participatory GMB for improvements in adolescent mental health and changes in wider social determinants [9].

## Discussion

### The application of group model building

This paper outlines the objectives and methods of a series of participatory group model building sessions within the Kailo programme. The aims—to engage with and collaborate alongside key stakeholders, to explore deeper into the social determinants of poor mental health and to gain a comprehensive insight into systemic behaviours—align with the methods described earlier. Participants are selected to ensure commitment from essential local partners and to include a spectrum of viewpoints, especially those often overlooked.

The methods draw from established group model building scripts, leading to a series of outcomes that culminate in a theoretical model of drivers for the opportunity area, represented as a causal loop diagram. The chosen activities aim to bring forth voices that are underrepresented in existing literature, particularly young people, offering a broader and more detailed understanding of the issue. This protocol advances research and tools in the field of community-based system dynamics, acknowledging the value of a participatory approach to modelling complex systems [26]. Consequently, this protocol supports researchers, designers and community leaders in both utilization of group model building within design processes and the engagement of young people in the method.

### Novelty of approach and justification (for writing a protocol)

This paper enhances the existing literature on group model building approaches by outlining their application in a novel context: focusing on social determinants of health and involving young people through group model building to shape co-designed local responses.

Furthermore, it presents a new approach to assessing the success of implementation by concentrating on the mechanisms through which group model building operates. This is informed by realist evaluation methodologies, which distinguish between what is essential when adapting interventions to a new context, what alterations are needed for that context and what changes are permissible during delivery.

### Measures of success core/flex

The participatory and evolving nature of this work necessitates adaptability to foster trust, psychological safety and understanding amongst participants involved in the group model building sessions. Consequently, the core research team might adjust the protocol to cater to these emerging needs. To promote psychological safety, the individual support needs of participants will be assessed during one-on-one sessions prior to the group model building workshops. Such assessments might lead to necessary changes in the workshop protocol. Moreover, as the sessions will occur in two distinct settings (Newham and Northern Devon), additional adaptations might be needed to align with the capabilities of local resources and stakeholders. All changes to the research protocol will be meticulously documented and communicated in subsequent findings.

Given that group model building methods are often customized to a specific project or community, evaluating the success of a CBSD process presents challenges [16]. This paper proposes using a realist evaluation framework, which highlights the importance of retaining mechanisms that contribute to successful implementation while allowing flexibility in tailoring the method to specific contexts or in response to emerging needs [42]. The key mechanisms include consensus building, commitment to action and the exposure of power dynamics.

### Limitations and risks

A key limitation of this method is that the causal loop diagrams and areas for intervention exploration do not fully represent the broader population. Consequently, the dynamics, interventions and leverage points identified in one setting might not be relevant in others. While this can be seen as a limitation, it also highlights the strength of the approach, offering in-depth insights relevant to specific community contexts.

The intensive engagement required for the group model building process limits the number of participants who can attend the sessions. Moreover, the recruitment of these select participants heavily relies on local community partners and their existing relationships with young individuals and community venues. In this way, it’s challenging for the group to truly reflect the diversity of the localities, potentially biasing the overall research findings [21].

While community-based system dynamics prioritizes training and empowering group model building participants, the technical nature of the activities and the conceptualization of variables linked in feedback structures can deter engagement. This concentrates significant influence with the modeller, who has a deeper expertise in the language of causal loop diagrams and conceptual modelling [28].

Additionally, system dynamics as a methodology operates on a core assumption that feedback mechanisms inherently outweigh even strong linear causal relationships [28]. This poses a risk that the models generated might overemphasize feedback structures at the expense of linear relationships. Additionally, CLDs often reduce feedback structures to look the same despite variations in loop dominance. This means that certain loops and structures of the system maps that have greater or lesser influence may be treated the same within the prioritization of action areas. Finally, CLDs developed through group model building are challenging to validate (ibid), even when compared to behaviour-over-time graphs created by the same group, given the difficulty in determining which feedback loop prevails at specific points in the map.

## Conclusions

This paper introduces a participatory group model building approach aimed at understanding the systemic drivers of poor mental health among adolescents in Newham and Northern Devon. Drawing from existing literature on similar group model building sessions, it elaborates on the workshop designs and the activities to be employed, explaining how they align with the session’s objectives. The outcomes of this process are designed to guide the co-creation of innovative local interventions that address the social determinants of adolescent mental health. By adopting a systemically informed perspective from community-based system dynamics, these interventions should target the fundamental drivers of the identified opportunity areas and aim to alter the underlying mental models of key stakeholders responsible for rolling out the devised interventions. Additionally, this paper enriches the literature by incorporating a framework from feasibility and implementation evaluation. This framework will scrutinize implementation and adaptation based on the delivery of the session’s core components, noting any modifications and their reasons.

## Data Availability

No datasets were generated or analysed during the current study.

## Appendix

### Session materials

- Flip chart paper
- Makers, pens
- A4 paper
- Adhesive

### Facilitation guides

#### Group model building session 1

Total time: 120 min (150 including break for food).

Materials needed: Information sheets, white board, flip chart paper, pens, A3 paper, tape.

**Table:**

Timing	Activity	Aim(s)	Materials	Group formations	Facilitators
15 min	Check-in and introductions	Recap of group agreement from previous sessions Reminder of group agreement and expectations Opportunity to flag anything important at the beginning of the session	Information from previous session Space for writing reminders of group expectations (either paper, whiteboard, screen, etc.)	Large group	Meeting convenor (site lead)
5 min	Aims and expectations	Review agenda for the day, open the space for any (quick clarifying) question	Agenda written somewhere so people have a reference for the day	Large group	Modeler facilitator
15 min	Session 2 recap	Remind group about learning surrounding the opportunity area from session 2 Allow space for additions from small circle	Inputs from previous session (printed)	Large group	Site lead and community facilitator
5 min	*Energizer activity*				Community facilitator
5 min	Systems thinking refresher	Reminder of the purpose of these activities Considerations for sharing lived experience (personal and communal) Introduction to core system thinking concepts	Systems thinking handout	Large group	Modeller facilitator
20 min	Trends over time	Identify/uncover historical trends related to the opportunity area. Reflect on how things have changed (to inform what makes something improve or get worse) Theme the trends Collectively decide upon which trends best encapsulate the relationship between the opportunity area and young peoples’ wellbeing	Paper and pens/markers, tape/adhesive for walls	5 min in in large group 10 min in small groups 5 min sharing back to large group and voting on trend	Modeller facilitator, supported by the community facilitators and site lead in small groups
25 min	Identifying factors (variable elicitation)	Identify factors related to the opportunity area, ideally specifically related to the trends over time (what makes in increase, decrease, or stay the same) Develop “word bank” for future exercises	Paper and pens/markers, tape/adhesive for walls	Large group—individual reflection (5–10 min) 15–20 min building out the wall	Modeller facilitator, community facilitator
*10 min (to be added within the session when needed)*	*Energizer activity*			Large group	Community facilitator
20 min	Connection circles	Introduce group to system dynamics concepts needed for the system mapping exercise in the next session Start to identify relationships between variables	Flip chart paper, markers	Small groups	Model facilitator
30 min	Food and check out activity			Large group	Meeting closer (site lead)

#### Group model building session 2

Total time: 120 min (150 including break for food).

Materials needed: Print outs from last session, white board (if possible), flip chart paper, pens, A3 paper, A4 paper, tape.

**Table:**

Timing	Activity	Aim(s)	Materials	Group formations	Facilitators
20 min	Check-in, welcome, review of aims and expectations	Recap of group agreement Opportunity to flag anything important at the beginning of the session Team-building amongst the group Open space for asking questions about the day	Group agreement print out Agenda written somewhere so people have a reference for the day	Large group	Meeting convenor (site lead)
20 min	GMB Session 1 recap	Remind group about learning surrounding the opportunity area from the last session System specialist reflections shared Allow space for additions from small circle group	Inputs from the previous sessions (printed)	Large group	Modeller facilitator, community facilitator
5 min	*Energizer activity*				Community facilitator
15 min	Introduction to feedback thinking and systems mapping	Introduction to feedback thinking Preparing everyone for the CLD exercise Ground the CLDs in core system theme(s) as defined by the group	White board, paper, systems thinking cheat sheet (printed)	Large group	Modeller facilitator
50 min	Causal loop diagrams (CLD)	Develop causal loop diagrams related to the opportunity area Explore connections between factors identified as related to the OAs	Large sheets of paper, markers/pens, white boards if possible	Small groups (3–4 people)	Modeller facilitator, supported by the community facilitators and site lead in small groups
10 min	Reflections and next steps	Reflect back learning from the exercise in small groups with the larger group Close out the activity and describe the work that will take place for the next session (modeller to combine the system maps)		Large group	Modeller facilitator
30 min	Food and check out activity			Large group	Meeting closer (site lead)

#### Group model building session 3

Total time: 120 min (150 including break for food).

Materials needed: Print outs from last session, white board (if possible), flip chart paper, pens, A3 paper, tape.

**Table:**

Timing	Activity	Aim(s)	Materials	Group formations	Facilitators
20 min	Check-in, welcome, review of aims and expectations	Recap of group agreement Opportunity to flag anything important at the beginning of the session Team-building amongst the group Open space for asking questions about the day	Group agreement print out Agenda written somewhere so people have a reference for the day	Large group	Meeting convenor (site lead)
10 min	Introduction to refined CLD	Review of the causal loop diagrams created in the session 2 (alongside any refinements made by the modelling team) System specialist reflections shared	Refined CLDs printed on large sheets of paper	Large group	Modeler facilitator, community facilitator
45 min	CLD review exercise	Reviewing CLD in small groups Allow space for additions and refinements from the small circle	Refined CLD printed	Small groups	Modeller facilitator, supported by the community facilitators and site lead in small groups
5 min	*Energizer activity*				Community facilitator
30 min	Places to intervene	Introduction to leverage points and places to intervene in the system Identification of action areas related to the opportunity areas	White board, paper Printing of the CLDs	Small groups	Modeller facilitator, supported by the community facilitators and site lead in small groups
10 min	Questions and reflections	Space to close out and reflect as a large group about the exercises	System maps with identified areas of intervention	Large group	Model facilitator
30 min	Food and check out activity			Large group	Meeting closer (site lead)

## Abbreviations

CBSD: Community-based system dynamics
CLD: Causal loop diagram
GMB: Group model building
OA: Opportunity areas

## References

[1] The Children’s Society. The good childhood report 2020. London: The Children’s Society; 2020.

[2] Newlove-Delgado T, Marcheselli F, Williams T, Mandalia D, Davis J, Mcmanus S, Savic M, Treloar W, Ford T. Mental health of children and young people in England 2022. Leeds: NHS Digital; 2022.

[3] Department for Health and Department for Education. Transforming Children and Young Peeple’s Mental Health Provision: a Green Paper. December; 2017.

[4] Gunnell D, Kidger J, Elvidge H. Adolescent mental health in crisis. BMJ. 2018;361: k2608. 29921659 10.1136/bmj.k2608

[5] Richter D, Dixon J. Models of mental health problems: a quasisystematic review of theoretical approaches. J Ment Health. 2022;32:396–406. 35014924 10.1080/09638237.2021.2022638

[6] Foulkes L. Losing our minds: what mental illness really is—and what it isn’t. St. Martin’s Publishing Group; 2021.

[7] Marmot M, Allen J, Boyce T, Goldblatt P, Morrison J. Health equity in England: the marmot review 10 years on. London: The Health Foundation; 2020. 10.1136/bmj.m693 32094110

[8] Farrell A, Hu M, Evbuoma EI, Liem W, Ballard E. Characteristics of complex problems methods brief series 102: systems thinking foundations. St. Louis: Social System Design Lab; 2021.

[9] Hobbs T, et al. Kailo: a systemic approach to addressing the social determinants of young people’s mental health and wellbeing at the local level. Wellcome Open Research; 2023. 10.12688/wellcomeopenres.20095.1 PMC11126905 38798997

[10] Kennedy L, March A, Harris J, Allen K, Hanley Santos G, Davies K, Malhotra T, Joshi K, Hobbs T, Fonagy P, Pilling S, Berry V (forthcoming) How does Kailo work to improve adolescent mental health? A developmental realist evaluation protocol.

[11] Santana de Lima E, Rehill N, Preece C, Harris J, Hobbs T, Fonagy P. Codesigning strategies to support young people’s mental health in Newham and Northern Devon; forthcoming.

[12] Compton MT, Shim RS. The social determinants of mental health. Clin Synth. 2015;13(4):419–25.

[13] Hartas D. The social context of adolescent mental health and wellbeing: parents, friends and social media. Res Pap Educ. 2021;36(5):542–60.

[14] Dreier L, Nabarro D, Nelson J. Systems leadership for sustainable development: strategies for achieving systemic change. Harvard Kennedy School; 2019.

[15] Forrester JW. Policies, decisions, and information sources for modeling. Eur J Oper Res. 1992;59(1):42–63.

[16] Hovmand PS. Community based system dynamics. New York: Springer; 2014.

[17] Jagosh J. Realist synthesis for public health: building an ontologically deep understanding of how programs work, for whom, and in which contexts. Annu Rev Public Health. 2019;40:361–72. 30633712 10.1146/annurev-publhealth-031816-044451

[18] Saul JE, Willis CD, Bitz J, et al. A time-responsive tool for informing policy making: rapid realist review. Implement Sci. 2013;8:103. 24007206 10.1186/1748-5908-8-103 PMC3844485

[19] Shamrova DP, Cummings CE. Participatory action research (PAR) with children and youth: An integrative review of methodology and PAR outcomes for participants, organizations, and communities. Children Youth Serv Rev. 2017;81:400–12. 10.1016/j.childyouth.2017.08.022. (**ISSN 0190-7409**).

[20] Finegood DT. The importance of systems thinking to address obesity. Nestle Inst Workshop Ser. 2012;73:123–37 (**discussion 139-41**). 10.1159/000341308 23128771

[21] Savona N, Macauley T, Aguiar A, Banik A, Boberska M, Brock J, Brown A, Hayward J, Holbæk H, Rito AI, Mendes S, Vaaheim F, van Houten M, Veltkamp G, Allender S, Rutter H, Knai C. Identifying the views of adolescents in five European countries on the drivers of obesity using group model building. Eur J Pub Health. 2021;31(2):391–6. 33608719 10.1093/eurpub/ckaa251 PMC8071593

[22] Savona N, Brown A, Macauley T, Aguiar A, Hayward J, Ayuandini S, Habron J, Grewal NK, Luszczynska A, Mendes S, Klepp KI, Rutter H, Allender S, Knai C. System mapping with adolescents: using group model building to map the complexity of obesity. Obes Rev. 2022;24(S1):e13506. 10.1111/obr.13506. 36825369 10.1111/obr.13506

[23] Langellier BA, Kuhlberg J, Ballard EA, Slesinski SC, Stankov I, Gouveia N, Meisel JD, Kroker-Lobos MF, SarmientoO L, Caiaffa WT, Diez Roux AV, SALURBAL Group. Using community-based system dynamics modeling to understand the complex systems that influence health in cities: The SALURBAL study. Health Place. 2019;60: 102215. 31586769 10.1016/j.healthplace.2019.102215 PMC6919340

[24] Reumers L, Bekker M, Hilderink H, Jansen M, Helderman JK, Ruwaard D. Qualitative modelling of social determinants of health using group model building: the case of debt, poverty, and health. Int J Equity Health. 2022;21:72. 35590354 10.1186/s12939-022-01676-7 PMC9118602

[25] Currie DJ, Smith C, Jagals P. The application of system dynamics modelling to environmental health decision-making and policy—a scoping review. BMC Public Health. 2018;18:402. 29587701 10.1186/s12889-018-5318-8 PMC5870520

[26] Freebairn L, Occhipinti J, Song YJC, Skinner A, Lawson K, Lee GY, Hockey SJ, Huntley S, Hickie IB. Participatory methods for systems modelling of youth mental health: implementation protocol. JMIR Res Protoc. 2022;11(2): e32988. 35129446 10.2196/32988 PMC8861863

[27] Vennix JAM. Group model building: facilitating team learning using system dynamics. Chichester: Wiley; 1996.

[28] Barbrook-Johnson P, Penn AS. Causal loop diagrams. In: Barbrook-Johnson P, Penn AS, editors. Systems mapping. Cham: Palgrave Macmillan; 2022.

[29] Design Council. Framework for innovation; 2024. https://www.designcouncil.org.uk/our-resources/framework-for-innovation/. Accessed Jan 2024.

[30] Wynn D, Maier A. Feedback systems in the design and development process. Res Eng Design. 2022;33:273–306.

[31] Hovmand PS, Andersen DF, Rouwette E, Richardson GP, Rux K, Calhoun A. Group model building “scripts” as a collaborative tool. Syst Res Behav Sci. 2012;29:179–93.

[32] Werner K, Starnold G, Crea TM. Using a community-based system dynamics approach for understanding inclusion and wellbeing: a case study of special needs education in an eastern African refugee camp. Conflict Health. 2021;15(1):58. 34301295 10.1186/s13031-021-00390-5 PMC8299607

[33] Siokou C, Morgan R, Shiell A. Group model building: a participatory approach to understanding and acting on systems. Public Health Res Pract. 2014;25(1): e2511404. 25828443 10.17061/phrp2511404

[34] Vennix JA. Group model-building: tackling messy problems. Syst Dyn Rev. 1999;15(4):379–401.

[35] Rouwette EA, Vennix JA, Mullekom TV. Group model building effectiveness: a review of assessment studies. Syst Dyn Rev. 2002;18(1):5–45.

[36] Kumar P, Chalise N, Yadama GN. Dynamics of sustained use and abandonment of clean cooking systems: Study protocol for community-based system dynamics modeling. Int J Equity Health. 2016;15(1):70. 27113743 10.1186/s12939-016-0356-2 PMC4845479

[37] Luna-Reyes LF, Martinez-Moyano IJ, Pardo TA, Cresswell AM, Andersen DF, Richardson GP. Anatomy of a group model-building intervention: building dynamic theory from case study research. Syst Dyn Rev. 2006;22(4):291–320.

[38] Kim D. System archetypes I: diagnosing systemic issues and designing high leverage interventions. Pegasus Communications Inc; 2000.

[39] Zellner M, Massey D, Rozhkov A, Murphy JT. Exploring the barriers to and potential for sustainable transitions in urban-rural systems through participatory causal loop diagramming of the food–energy–water nexus. Land. 2023;12(3):551.

[40] Zucca C, McCrorie P, Johnstone A, Chambers S, Chng NR, Traynor O, Martin A. Outdoor nature-based play in early learning and childcare centres: Identifying the determinants of implementation using causal loop diagrams and social network analysis. Health Place. 2023;79: 102955. 36565541 10.1016/j.healthplace.2022.102955

[41] Moore G, Campbell M, Copeland L, Craig P, Movsisyan A, Hoddinott P, et al. Adapting interventions to new contexts—the ADAPT guidance. BMJ. 2021;374: n1679. 34344699 10.1136/bmj.n1679 PMC8329746

[42] Evans RE, Moore G, Movsisyan A, The ADAPT Panel, et al. How can we adapt complex population health interventions for new contexts? Progressing debates and research priorities. J Epidemiol Community Health. 2021;75:40–5. 32981892 10.1136/jech-2020-214468 PMC7788480

[43] Andersen DF, Richardson GP. Scripts for group model building. Syst Dyn Rev. 1997;13(2):107–29.

[44] Ford DN. A system dynamics glossary. Syst Dyn Rev. 2019;35(4):369–79.

[45] Hovmand PS, Kraus A. Creating causal loop diagram from connection circles. Scriptapedia. [web resource]; 2013. https://en.wikibooks.org/wiki/Scriptapedia/Creating_Causal_Loop_Diagram_from_Connection_Circles. Accessed 28 Apr 2023.

[46] Meadows D. Leverage points: places to intervene in a system. Hartland: The Sustainability Institute; 1999.

